# Emergency preparedness in the central sterile supply department: a multicenter cross-sectional survey

**DOI:** 10.1186/s12873-024-01053-3

**Published:** 2024-07-29

**Authors:** Jiawei Liu, Fengliu Gui, Mengmeng Zhang, Hui Chen

**Affiliations:** West China School of Nursing/West China Hospital, Sichuan University, Guoxuexiang No. 37, Chengdu, Sichuan China

**Keywords:** Central Sterile Supply Department, Emergency preparedness, Emergency drill, Nurse, Emergency attitude, Emergency capacity, Cross-sectional survey

## Abstract

**Objective:**

To investigate the current situation of emergency preparation and emergency drill in the CSSD, and analyze its influence on the nurses’ emergency attitude and ability.

**Methods:**

This study employed a multicenter stratified sampling method, conducted from January to June 2023 using the online survey, participants completed the general data, emergency preparedness and drill questionnaire, public health emergency response questionnaire and emergency capacity scale. An independent samples t test or Kruskal-Wallis test was used to analyse differences in nurses’ emergency capacity and attitudes.

**Results:**

The data from 15 provinces 55 hospitals in China. Overall, 77.58% of participants’ institutions set up emergency management teams, 85.45% have an emergency plan and revise it regularly. 92.12% store emergency supplies. All survey staff participated in the emergency drill, which predominantly consisted of individual drills (51.52%), with 90.30% being real combat drills, 49.09% of participants engaging in drills every quarter, and 91.52% of the drill’s participants exceeding 50%. The respondents’ emergency attitude score was (29.346 ± 6.029), their emergency ability score was (63.594 ± 10.413), and those with rescue experience showed a more positive attitude (Z = -2.316, *P* = 0.021). Different titles, education levels, rescue experience and the frequency of emergency drill affected the emergency rescue ability of the respondents (*P* < 0.05).

**Conclusions:**

Most medical institutions establish emergency management systems and plans, yet the content lacks geographical specificity.The duration and participation of emergency drills are high, but the effectiveness of the drills needs to be further improved, and the response capacity and attitudes of CSSD nurses are low. It is recommended that agencies develop comprehensive and targeted contingency plans to strengthen the inspection and evaluation of team strength, equipment and safeguards against the contingency plans, so as to ensure that the measures mandated by the contingency plans can be implemented promptly after the emergency response is initiated.

**Supplementary Information:**

The online version contains supplementary material available at 10.1186/s12873-024-01053-3.

## Introduction

Emergency refers to the sudden occurrence of major infectious diseases, serious mass diseases, major food and occupational poisoning, and other events that cause or may cause serious damage to public health [1]. Public health emergencies have the characteristics of outbreak, harmfulness, concealment and complexity, which not only threaten the safety of life and property but can also cause serious economic losses [2]. In recent years, with the development of industry, the economy and society, the continued development of natural resources, and changes in climate and environment, global emergencies have increased significantly. Statistics from the global disaster data platform show that from August 22,2022 to August 22,2023, 968 disasters occurred around the world, affecting 2630,456,500 people and resulting in a cumulative economic loss of 597,7150,000 US dollars [3]. China is among the countries with the most t natural disasters in the world. Disasters occur in many forms, are widely distributed, have a high frequency, and cause heavy casualties.Safety at work is still in a period of escalation, all types of safety risks are interwoven, and production safety accidents are still a concern [4].

Equally important, according to the World Health Organization, is the provision of emergency relief, disaster preparedness, and preventive measures [5]. As a key department for nosocomial infection management, the Central Sterile Supply Department (CSSD) is responsible for cleaning, disinfecting and sterilizing all reusable diagnostic and therapeutic instruments, and items in hospitals [6]. In the event of an emergency, while protecting its own safety, the CSSD also needs to shoulder the function of material support. Medical equipment and other materials should be sent to the user service as soon as possible after a major natural disaster to reduce casualties or mass chaos. In the face of unknown and complex external environments, CSSD emergency management poses new challenges. CSSDs also have a high risk of internal emergencies when faced with external contingencies. The CSSD is a key energy use department in the hospitals and has a 100% special equipment allocation rate, and risks such as fire and gas leaks are very likely to occur if it is poorly maintained during use [7]. CSSD is also the concentration of various disinfection and sterilizing chemicals. Chemical disinfectants are typically flammable and explosive and cause severe damage to personnel following leaks. CSSD simultaneously stores various flammable packaging materials; in the case of fire, if rescue is not timely and action is not taken, the golden hour of rescue will be missed, leading to further expansion of the accident. Thus, strengthening the emergency management capability of CSSD is imperative.

Catastrophic events can occur anywhere, and at any time, they are emergencies. It has overwhelmed the resources of disaster areas or places, leading to great human suffering and severe economic loss [8]. Disaster preparedness refers to the measures taken before the disaster occurs to minimize the loss of life. Effective emergency rescue capability, high awareness of self-rescue and mutual rescue, and effective evacuation can greatly reduce accident-related casualties. An emergency drill, as a key to testing the contingency plan and training the team, is an important way to improve emergency response capacity and is the key link in emergency management. The term emergency drill refers to setting the scene of work and the management system as realistic or as possible for trainees to complete a series of tasks as required to improve the capacity for emergency or emergency response or the level of emergency operation. Studies show that emergency drills have the potential to improve risk discovery and emergency handling capability by 11% and 13%, respectively [9]. Therefore, there is an urgent need to strengthen emergency drills to avoid or reduce the risks and hazards caused by major natural disasters.

In the emergency rescue phase of public health emergencies, nurses are one of the largest groups providing health services. The speed and effectiveness of emergency rescue work in disaster areas are directly influenced by the emergency rescue attitude and the emergency response capability of the nursing staff and have a large impact on and contribution to disaster prevention, reduction, and management in the aftermath of disasters. However, studies show that ED nurses are at only moderate levels of disaster preparedness, and in the face of emergencies, most carers have little experience with emergency response [10]. At present, there are some studies on disaster preparedness. The subjects of these studies included ICU [11], community [12], and other professional nursing students and nurses [13]. In the context of these previous studies, we focus on the Chinese mainland, including Sichuan Province, Hubei Province, and Shanghai. These published studies, however, have limitations such as unclear research methods and incomplete data outcomes. Given that the CSSD nurses are generally younger, have lower awareness of self-protection and medical risks, and lack professional disaster training, it is urgent to effectively prepare and respond to emergencies. Proper management of equipment, protection of medical personnel, and reduction of hospital property loss are pressing issues that need to be addressed. Therefore, this study investigates the situation of Chinese CSSD emergency drills and the emergency capacity and attitude of nurses to implement the policy of “safety first, prevention first”, standardize emergency management, enhance the ability to comprehensively deal with emergencies, and minimize property losses, environmental damage and social impact.

## Methods

### Design

In the present study, a cross-sectional, multicenter online survey was used from 2023.01 to 2023.06.

### Samples and setting

A stratified sampling approach was used to select 2 to 3 provinces and cities in East China, Central China, North China, Southwest China, Northwest China, and Northeast China. A minimum of 2 hospitals in each province and city were selected as research sites, and the CSSD nurses in each hospital were selected as the objects of research. The subject inclusion criteria included obtaining a qualification certificate from the practice nurse, having a minimum of 5 years of CSSD experience, being employed, and providing informed consent. The exclusion criteria were nurses who were involved in advanced study, specialized or rotated and nurses who were not aware of the status of their institution’s emergency drills. In the end, 55 hospitals and 165 CSSD nurses from 15 provinces and towns (Hubei, Anhui, Gansu, Yunnan, Jilin, Liaoning, Henan, Xinjiang Uygur Autonomous Region, Guizhou, Sichuan, Shanxi, Chongqing, Jiangsu, Shaanxi, Fujian) were enrolled in the study.

### The tools

The questionnaire was compiled in accordance with the Emergency Response Exercise Guide [14], which was published by the Emergency Management Office of The State Council of the People’s Republic of China, and data were obtained from the following variables: (a) individuals and their facilities: it consisted of 15 questions including sex, age, education, job title, rescue experience, hospital grade, type of hospital, and number of beds. (b) Emergency preparedness consists of 17 questions, such as emergency management team, emergency system, emergency process, emergency plan, emergency materials, and emergency management post responsibilities. (c) Emergency drill: It consists of 9 questions, such as the content of drill, the project of drill, the form of drill, the duration of drill, and the participation of emergency drill personnel. (d) Urgency response: The psychological urgency attitude scale for public health emergencies developed by Liu Yumei et al. [15] was adopted, consists of four dimensions, namely, personal psychological response, attitude toward personal training, personal expectation of disaster relief, and self-rating, with a total of ten items. All items were rated on a 5-level Likert scale ranging from “completely disagree” to “completely agree” with a Cronbach’s α coefficient of 0.84. A total score of ten to fifty was assigned, and the personal psychological response dimension was reverse scored. A higher score on the scale indicates better contingency attitudes. The mean item score was used as the criterion, and > 4, 3 ∼ 4, and < 3 were split into high, medium, and low levels of urgency attitudes, respectively. (e) Emergency response capacity: The scale of emergency response capacity for public health emergencies was adopted [16]. The questionnaire consisted of 18 items in three dimensions, namely, emergency knowledge learning ability, first-aid ability and comprehensive ability. A 5-level Likert scale scoring method was adopted, and each dimension was “well done, well done, average done, poorly done, and very poorly done” with 5, 4, 3, 2, and 1 points, respectively. Eighteen to ninety total points were obtained, and the mean score of the items was set as the criterion for judging. A score of < 3 points indicated low emergency capacity, and a score of 3 to 4 points indicated moderate emergency capacity. A score of > 4 indicates high emergency response capability. This scale had a Cronbach’s α value of 0.950.

Six leaders in the CSSD area were asked to evaluate the questionnaire, and the questionnaire was modified based on their views. Ten managers of CSSD in Sichuan Province were then asked to complete a preexperiment to assess the feasibility and approximate completion time of the questionnaire and to further optimize the questionnaire.

### Data collection

In this study, an electronic questionnaire was used to collect data. The questionnaire links and two-dimensional code were generated on the Questionnaire Star platform (https://www.wjx.cn/). Each participant received the survey via WeChat or email, and each participant was required to submit the survey within 2 weeks of receiving it. The questionnaire explained the purpose, meaning, and method of completion of the survey using unified guide words, and subjects completed and submitted the questionnaire independently. The questionnaire requires all items to be answered, and an electronic device can answer only once. Following receipt of the questionnaire, members of the research team double checked the quality of the questionnaire, and in the event of ambiguity, participants were contacted by telephone to check data for accuracy. The checklist included the following indicators: (a) no missing items; (b) fidelity to facts; and (c) no reverse response to the same question. Any questionnaire with the same items and less than 2 min of fill-in time was considered invalid and excluded.

### Data analysis

SPSS version 25.0 (IBM Corp, Armonk, NY) statistical software was used for data analysis. Descriptive analysis of the hospitals, demographic characteristics of the study subjects, emergency preparedness status, emergency attitude, and emergency response capacity revealed that quantitative data were expressed as the mean or median and interquartile range (IQR), whereas classified data were expressed as the frequency and corresponding ratio. Independent samples t tests or Kruskal‒Wallis rank sum tests was used to analyze differences in the emergency attitudes and emergency capacities of the CSSD nursesfor the different artificial stomatological features and emergency drills. A value of *P* < 0.05 was considered to indicate statistical significance.

### Ethical considerations

This study was approved by the Biomedical Ethics Committee of West China Hospital, Sichuan University (No. 2023 − 673). Since the study was an online survey, permission was obtained from all respondents via signed electronic informed consent. Participation in this study was voluntary.

## Results

### Demographic data of subjects and general hospital information

Survey data were collected from 55 hospitals and 165 CSSD nurses across 15 provinces in mainland China and were used in this study. Among the participants who completed the survey, 95.15% were male, 4.85% were female, approximately 48.48% were 41–50 years old, most of them (52.73%) were nurse-in-charge, 59.39% were head nurses, and 73.4% had rescue experience. The number of CSSD nurses in various medical institutions was mainly in the range of 30–49(23.64%) (see Table 1).

**Table 1:** Demographic data of the participants and general hospital information

Demographic characteristics	category	Frequency	percentage
Gender	man	8	4.85%
	woman	157	95.15%
Age(years)	< 30	17	10.30%
	30–40	47	28.48%
	41–50	80	48.48%
	> 50	21	12.73%
Professional titles	nurse	5	3.03%
	primary nurse	20	12.12%
	nurse-in-charge	87	52.73%
	cochief superintendent nurse	49	29.70%
	chief superintendent nurse	4	2.42%
Job title	Head nurse	98	59.39%
	Area leader	20	12.12%
	other	3	1.82%
	none	44	26.67%
level of education	Technical secondary school (high school) and below	3	1.82%
	Junior college	22	13.33%
	Undergraduate course	137	83.03%
	Master’s degree or above	3	1.82%
rescue experience	yes	121	73.4%
	no	44	26.6%
Working environment characteristics			
Hospital area	East China	18	10.91%
	Central China	43	26.06%
	North China	11	6.67%
	Northwest China	22	13.33%
	Southwest China	51	30.91%
	Northeast China	20	12.12%
Hospital grade	Secondary hospital	22	13.33%
	tertiary hospitals	143	86.67%
Types of hospitals	polyclinic	139	84.24%
	maternal and child care service center	8	4.85%
	mental health center	2	1.21%
	hospital of traditional Chinese medicine	14	8.48%
	other	2	1.21%
Number of beds	< 200	1	0.61%
	200–299	3	1.82%
	300–499	15	9.09%
	500–799	39	23.64%
	800–1200	28	16.97%
	> 1200	79	47.88%
Daily operation volume	< 50	69	41.82%
	50–99	39	23.64%
	100–300	48	29.09%
	> 300	9	5.45%
Number of CSSD staff	> 100	1	0.61%
	80–100	3	1.82%
	50–79	28	16.97%
	30–49	39	23.64%
	< 30	94	56.97%

### Emergency preparedness

**Fig. 1:**
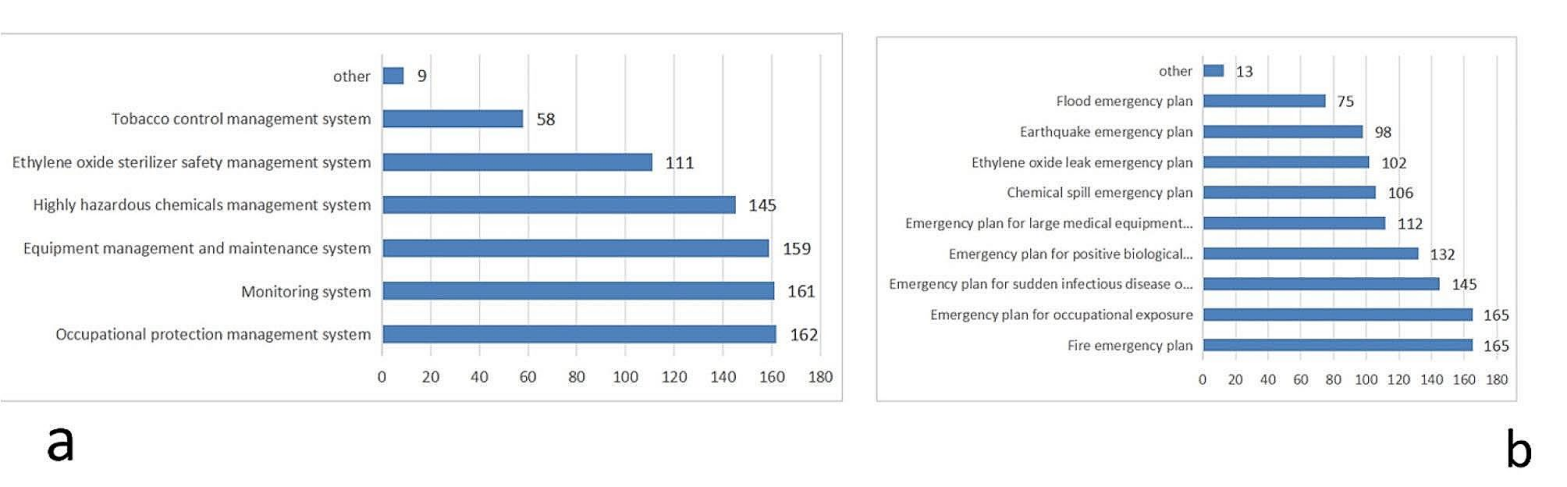
Frequency of emergency system and emergency plans. (**a**) Frequency of different types of emergency management systems. (**b**) Frequency of different types of emergency plans

**Fig. 2:**
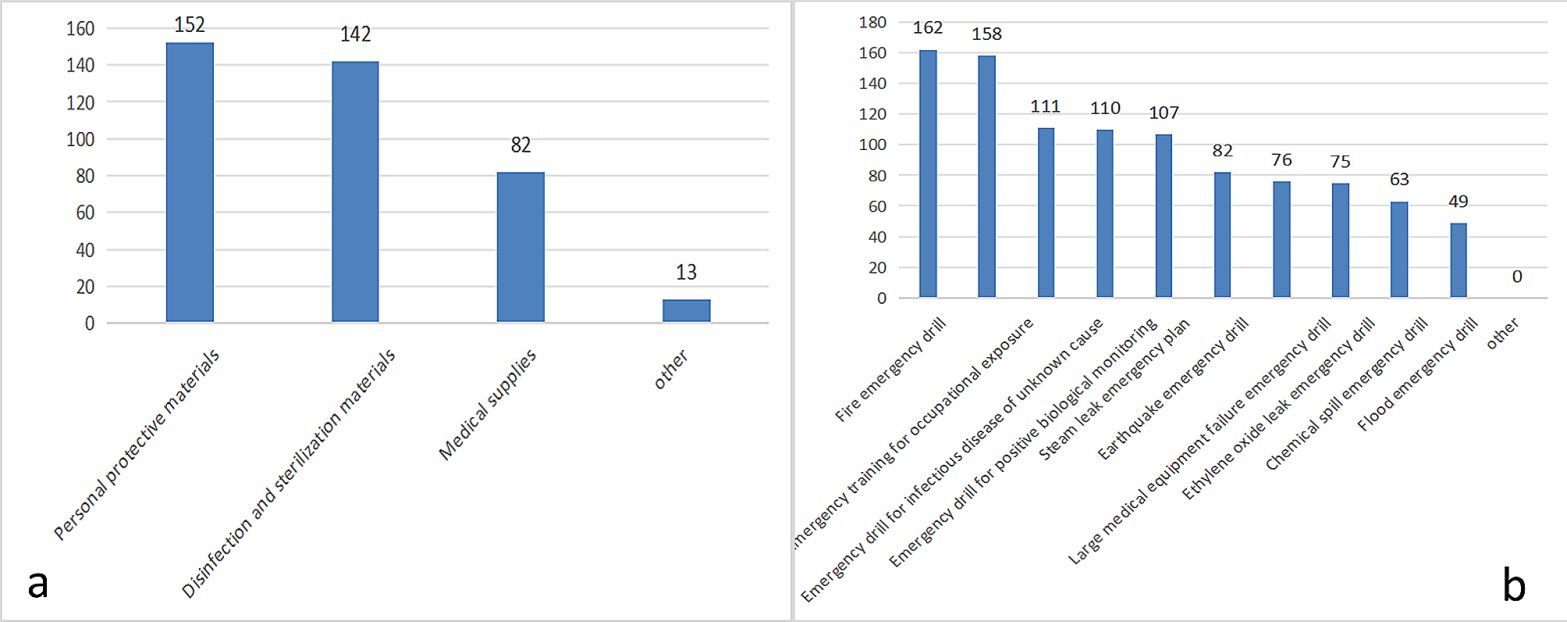
Frequency of emergency supplies and emergency drills. (**a**) Frequency of different types of emergency supplies plans. (**b**) Frequency of different types of emergency drills

**Fig. 3:**
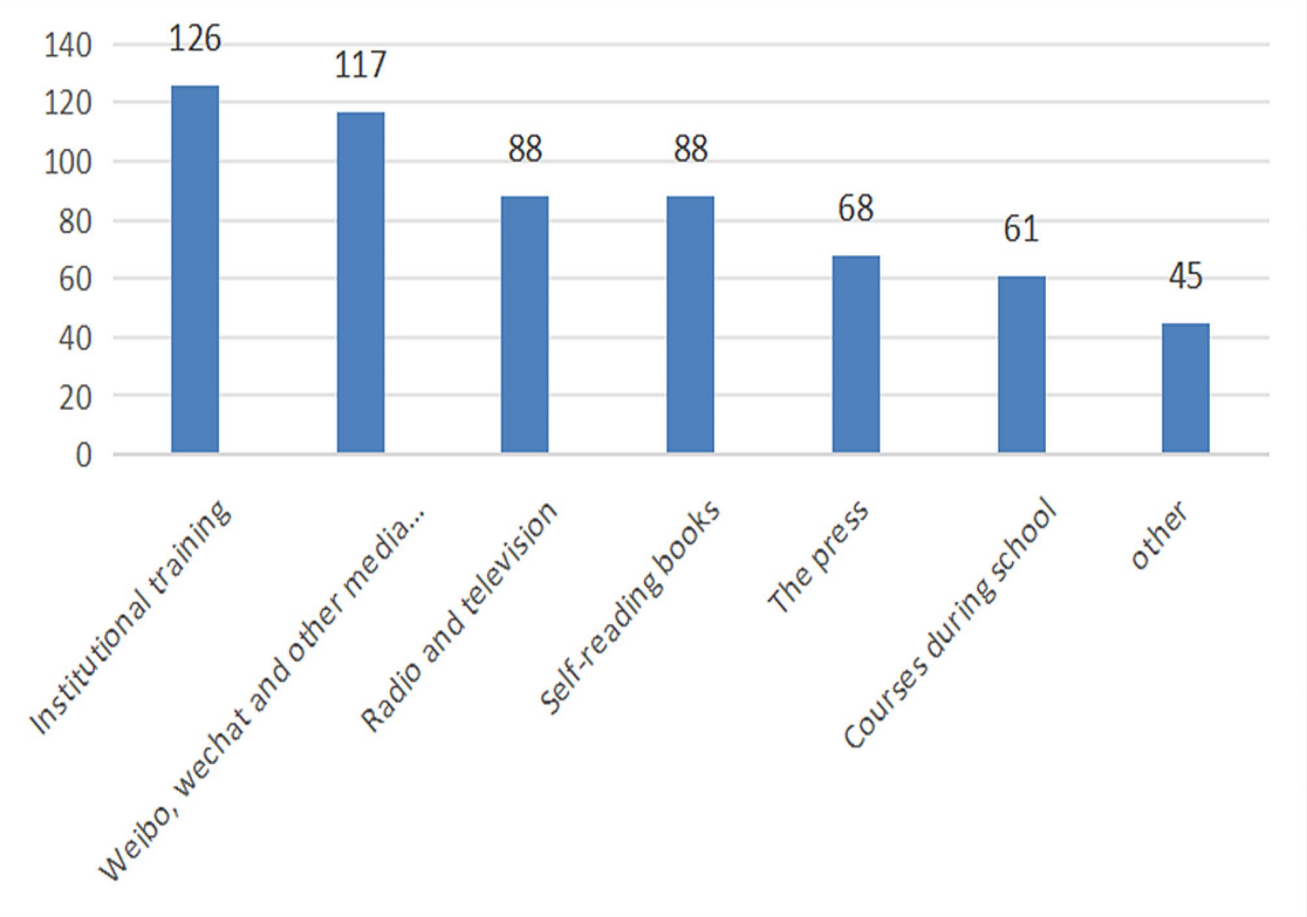
Frequency of different ways of acquiring knowledge

Among all participants, 77.58% set up emergency management teams, 98.79% established emergency management systems, and 70.30% had clear responsibilities for emergency positions. All participant institutions have established contingency plans, with regular updates and revisions of 85.45%(see Fig. 1). A total of 92.12% of the participants stored emergency supplies, and 78.18% of the CSSD had emergency boxes. A total of 96.97% of the participants had attended emergency training, and 61.82% had regularly attended emergency training. All members of the surveying army took part in the emergency drill(see Fig. 2), urgent drill content single drill (51.52%), 90.30% of emergency drills are conducted in the form of Combat exercise, 49.09% in the investigator drill at a frequency of once/quarter, and organization-to-host form (64.85%), with 91.52% in the exercise staff participation process reaching over 50% (see Table 2).Institutional training (76.36%) is the main way to acquire knowledge(see Fig. 3).

**Table 2:** Characteristics of emergency training, emergency plans, emergency materials and emergency drilsl (*n* = 165)

	category		Frequency		percentage		category	Frequency	percentage
**Emergency management**									
Emergency management team	yes		128		77.58%	Emergency management system	yes	163	98.79%
	no		37		22.42%		no	2	1.21%
Job responsibilities for emergency events	yes		116		70.30%				
	no		49		29.70%				
**Emergency plan**									
Emergency plan	yes		165		100.00%	Update or revise emergency plans regularly	yes	141	85.45%
	no		0		0.00%		no	24	14.55%
**Emergency supplies**									
Reserve emergency supplies	yes		152		92.12%	Emergency kit	yes	129	78.18%
	no		13		7.88%		no	36	21.82%
**Emergency training**									
Emergency related training	yes		160		96.97%	Frequency of training	frequently	102	61.82%
	no		5		3.03%		occasionally	52	31.52%
							seldom	6	3.64%
							never	5	3.03%
**Emergency drill**									
Content of emergency drill	Single emergency drill		85		51.52%	Forms of emergency drills	Combat exercise	149	90.30%
	Multiple emergency drill		80		48.48%		Desktop walkthrough	16	9.70%
Frequency of emergency drills	1/every six months		31		18.79%	Organizational form	attend	51	30.91%
	1/every quarter		81		49.09%		Co-Sponsors	7	4.24%
	1/every year		28		16.97%		sponsor	107	64.85%
	1/month		25		15.15%				
Drill duration	< 15 min		15		9.09%	Degree of participation	< 30%Personnel participation	7	4.24%
	15–30 min		52		31.52%		30-50%Personnel participation	7	4.24%
	30–60 min		76		46.06%		50-70%Personnel participation	20	12.12%
	60–90 min		7		4.24%		70-90%Personnel participation	68	41.21%
	90–120 min		7		4.24%		Full participation of the department	63	38.18%
	> 120 min		8		4.85%				

### Emergency attitudes and emergency capabilities of CSSD nurses

The emergency attitude of the respondents was 29.346 ± 6.029, the emergency ability score was 63.594 ± 10.413, and those with rescue experience showed a more positive attitude (Z= -2.316, *P* = 0.021). Different job titles, levels of education, rescue experience, and frequency of emergency drills affected respondents’ ability to respond (*P* < 0.05) (see Table 3).

**Table 3:** Comparison of emergency attitudes and emergency abilities according to individual and institutional characteristics

	category	Frequency	Emergency attitude	Emergency capacity
Gender	man	8	32.625 ± 5.069	57.750 ± 11.696
	woman	157	29.178 ± 6.039	63.892 ± 10.297
Test and significance			t = 1.585	t = 1.636
			*P* = 0.115	*P* = 0.104
age	<30	17	30.823 ± 5.282	62.765 ± 8.113
	30–40	47	29.085 ± 7.183	59.0(55,70)
	40–50	80	59(55,70)	64.025 ± 10.661
	> 50	21	30(25.5,35.5)	63.762 ± 13.088
Test and significance			Z = 1.780	Z = 1.219
			*P* = 0.619	*P* = 0.748
The title of a professional post	nurse	5	36(24,36)	54(52,54)
	primary nurse	20	29(22,34.25)	59.5(53,66.5
	nurse-in-charge	87	30(26,36)	62(55,69)
	cochief superintendent nurse	49	28(25,34)	68(62,77)
	chief superintendent nurse	4	30.5(26,35)	71(70,72)
Test and significance			Z = 3.481	Z = 25.425
			*P* = 0.481	*P* < 0.001
Job title	Head nurse	98	29(25,35)	63.5(55,70)
	Area leader	20	26(24,33.25)	61(52.25,73)
	other	3	31.33 ± 8.08	54.67 ± 6.35
	none	44	32(26,36)	67(57.25,69.75)
Test and significance			Z = 3.926	Z = 4.796
			*P* = 0.270	*P* = 0.187
Educational level	Technical secondary school (high school) and below	3	27.33 ± 4.618	84 ± 0
	Junior college	22	32.5(25,36)	64(53.75,75)
	Undergraduate course	137	30(25,35)	63(55,70)
	Master’s degree or above	3	27.0 ± 1.73	62 ± 12.12
Test and significance			Z = 1.230	Z = 8.538
			*P* = 0.0746	*P* = 0.036
Rescue experience	yes	121	30(25,36)	65(58,70.5)
	no	44	27.136 ± 6.99	58(53,69.5)
Test and significance			Z=-2.316	Z=-2.364
			*P* = 0.021	*P* = 0.018
Frequency of emergency drills	1/every year	28	28(25,35)	58(52.25,69)
	1/every six months	31	26(24,35)	58(53,66)
	1/every quarter	81	30(25,35)	63(55,71)
	1/month	25	28(25.50,36.5)	70(67,74)
Test and significance			Z = 1.178	Z = 26.245
			*P* = 0.758	*P* < 0.001

## Discussion

The purpose of this study was to investigate the current state of CSSD emergency preparedness and its impact on nurses’ emergency capacity and attitude. The survey results show that emergency preparedness among medical institutions at all levels is good but not specific enough. There is still a lack of emergency attitude and level of emergency capacity among nurses. To the best of our knowledge, this is the first study to investigate the state of emergency preparedness in CSSD. Therefore, this study is a key research outcome for the safety management of CSSD.

### CSSD emergency preparedness

In line with research by Zhang Suli et al. [17], it may be that with the frequent occurrence of emergencies, the state and the government are paying more attention to a variety of medical facilities and strengthening the management of hospital grade reviews and day-to-day inspection and supervision, prompting basic emergency systems and plan completion, as well as regular updates. In addition to the national environmental contingency plan such special arrangements, in other laws and regulations related to the environment, such as the People’s Republic of China’s environmental protection law, the law of emergency response of the People’s Republic of China, the national emergency plan for the environment, and the law of production safety of the People’s Republic of China, safety management regulations for hazardous chemical materials, interim management actions for environmental emergency plans, etc., also stipulated the formulation and implementation of relevant content. All of these factors have reinforced the formulation of emergency systems and plans. However, the content of the management system and plan is not comprehensive enough, and the specialized emergency system and plan are relatively comprehensive, but the emergency plan and natural disaster system focus primarily on fire, floods and earthquakes (see Fig. 1), ignoring that each region has its own regional features. This requires us to improve and enrich the emergency plan system on the basis of existing experience, such as supplementing the environmental events in recent years (water pollution in the basin, emergency solid waste pollution, etc.), which is also a supplement to China’s legal system. It is suggested that managers establish overall emergency plans and some special emergency plans. To further improve the scientific and systematic nature of the emergency plan system and to improve the pertinence and practicality of special emergency plans.

The construction of the emergency management team (77.58%) and emergency accountability (70.30%) is not perfect, planning and system after preparation is complete, planning clarifies each work requirement, and emergency disposal, emergency backup measures must also implement personnel from the department management team, staff and responsibilities that are not implemented, which will result in the implementation of the plan, analyses not an established management team and accountability. The main reason is subjective awareness of the plan is not high, the conscience of responsibility is not strong, the objective reason is inexperience, lack of manpower, and limited capital.

The primary purpose of contingency plan training is that the personnel who actually implement the contingency plan will have a detailed and complete understanding of the correct plan and for training corresponding emergency skills within the training process. In general, general emergency plan training content includes basic knowledge, basic skills, basic plan content, etc. The ultimate goal of training staff is to acquire the corresponding knowledge of the plan, to master the core competencies and to understand the core content of the plan. The survey results show that the proportion of urgent training facilities is often only 61.82%, and institutional training (76.4%) is the primary means of knowledge acquisition(see Fig. 3). Hospital managers must rapidly reinforce the publicity and education of emergency plans, execute the training plans, practice, establish the evaluation of the plan, review the mechanism, constantly improve emergency capacity, and constantly promote the construction of emergency systems. However, we should also strengthen the training of emergency preparedness staff to improve the quality of preparedness of the plan.

To provide prompt and effective emergency treatment in the event of a disaster, material preparation is an important component. In real work, time and labor are often wasted due to the preparation of various materials. There are different types of emergency boxes for various emergencies. An emergency box should have the characteristics of complete materials, be 100% efficient, have a fixed placement, and be delivered to the user in the shortest amount of time [18]. A number of researchers have explored the application of the emergency boxes in patients with epilepsy [19], transport of ICU patients [20], and chemotherapy patients [21] and have achieved remarkable results. In contrast, this study revealed that the preparedness rate of the CSSD emergency box was only 78.18%, suggesting that CSSD hospitals and managers should design practical, timely, and efficient emergency boxes based on the area and types of hazardous CSSD chemicals to ensure that CSSD staff saves time, manpower, speed and efficiency in responding to emergencies.

Modern nursing management is experiencing the development process from experience management to scientific management. In the event of an emergency, it should not only have a reasonable emergency process and a feasible emergency plan but also be fitted with emergency materials based on first aid. Emergency equipment is an important support in the emergency rescue and disposal process. The material reserve situation of the CSSD, as the key service in the logistics support of the hospital, has a significant impact on the emergency rescue work of the hospital. According to the survey results: CSSD emergency supplies were equipped with 92.12%, mostly personal protective materials (92.12%)(see Fig. 2), higher than research by Chen Boqiao [22]. The reason may be because this study used the national CSSD survey and most tertiary hospitals (86.87%), experience of emergency rescue and awareness of rescue are higher, and supplies with diplomas are also high. As part of the unified hospital arrangement and deployment, the CSSD should formulate an emergency medical supply catalog based on its own characteristics and the actual needs of the hospital, specifying reserve types and quantities and establishing and improving the reserve system for CSSD emergency medical supplies.

### CSSD emergency drill

Emergency plan exercises are the best way to test the rationality, scientificity, practicability and operability of emergency plans. Table 2 shows that CSSD emergency drills are mainly actual combat drills (90.30%). Actual combat drills put forward more specific and comprehensive requirements for various contents of the plan, which is better than desktop drills in terms of actual effect, but at the same time, they also require considerable resources and economic support, which is also a concern of various medical institutions in the exercise process. If the economic support is insufficient, then the emergency drill will not achieve the expected effect.

The CSSD hosted emergency drills primarily (64.85%) in terms of organizational form, the frequency of drills occurred more than once a quarter (49.09%), and some medical facilities conducted drills every 6 months or more that may only occasionally perform emergency drills to supplement the inspection of higher departments. This is irresponsible to both staff and hospital security.

The duration of the drill was more than 30 min, which was 59.39%. Medical facilities with more than 70% attendance reached 79.39% in terms of emergency drill attendance. This study demonstrated that the implementation of emergency drills by a variety of agencies is relatively high, and that supervision by government departments is effective, but drill content is focused and does not reflect differences between regions. Government departments or hospital emergency management teams are suggested to improve emergency department projects and at the same time oversee the actual contents of the emergency drills, with a focus on the feasibility of emergency plans, adequacy of emergency preparedness, coordination of emergency mechanisms, and timeliness of emergency disposition.

### CSSD nurses have positive attitudes and the ability to respond to emergencies

Based on the results of the survey, the emergency attitude score of the CSSD nurses was 29.346 ± 6.029, which was less than that in Liu Yan et al.‘s study [23]. Analyzing the reasons, it may be due to Liu Yan and others mainly investigating and researching the work of Wuhan’s tertiary hospitals. During the COVID−19 epidemic, medical workers in Wuhan faced significant work pressure, with almost all medical staff involved in emergency response. Therefore, their emergency response attitude during emergencies was more proactive. This is consistent with the research findings. CSSD personnel who have experienced emergencies show a higher emergency response attitude compared to those who have not.

We found that the emergency department capacity score of the CSSD nurses was 63.594 ± 10.413, which was lower than that in the study by Gu Xingqiong et al. [24] (70.38 ± 14.15 points). Reason analysis: It is possible that Gu Xingqiong et al. research subjects may be clinical nurses. Clinical nurses have more time to communicate with patients, are more skilled in a variety of nursing skills and have more rescue experience. Although CSSD nurses are not front-line clinical staff, they still have a responsibility and obligation to go to the front line of emergency rescue when an emergency arises. It is therefore suggested that hospital managers pay more attention to the CSSD special group and conduct relevant training and exercises to enhance their emergency response capabilities. The higher the professional title, the higher the level of education, and the greater the emergency capacity in response to public health emergencies. Individuals who have participated in emergency public health training have higher contingency capacity scores, which is consistent with findings from relevant studies [25]. Furthermore, previous studies show [26, 27] that emergency training and emergency drills are important steps in improving personnel’s capacity for emergency rescue, and the emergency rescue capacity of CSSD nurses can be enhanced by targeted training and the organization of regular emergency drills. Emergency drills were performed by all medical institutions in this study, and the duration and attendance of emergency drills was relatively high, but the emergency capacity of the CSSD nurses was still low. This shows that at present, there are more scripts, processes and “performance” prepared, and the effect of observation is good, but the effect on finding the real issues and seeing the real situation is bad. CSSD emergency drills are currently primarily based on fire emergency handling drills. Despite the large number of “performances”, there is no “practice” effect, reflected mainly in the fact that the actual combat effect cannot achieve the expectation, and the relevance is not strong. An emergency drill is an important step in increasing the team’s awareness of risk, enhancing the team’s emergency awareness, and optimizing the emergency plan. It is an important carrier for strengthening the team’s real combat capability but also an important way of finding problems and testing the true effectiveness of emergency rescue. An urgent drill should be practiced and should not become a formality.

### Limitation of study

There are a few limitations to this study. First, the current study utilized the convenience sampling method, and the number and grade of hospitals selected across the provinces were inconsistent. The number of nurses surveyed varied across hospitals, which may have contributed to selection bias and limited extrapolation. Second, the current study utilized an online questionnaire survey. Although the method of self-completion in the questionnaire has been a quality control in questionnaire design and postprocessing of data, measurement bias may still exist.

## Conclusions

CSSD nurses are not only first-line rescue personnel but also the primary force for logistical support. The most critical and pressing link for improving the emergency management of CSSD nurses is the emergency drill. The results of this study indicated that the emergency management system and medical facility plan preparation were satisfactory, but the content lacked regional characteristics. Although the time and attendance of emergency drills was high, the effect of drills still needed to be improved. CSSD nurses had poor emergency response capability and attitude. As a result, a comprehensive and focused emergency plan should be formulated, and inspection and evaluation of team strength, equipment, and materials, as well as safeguard measures, should be strengthened against the plan to ensure that the tasks and actions stipulated in the plan can be rapidly implemented after the emergency response has been initiated. Strengthening the publicity and training of the emergency plan and consistently improving the emergency capacity and attitude of CSSD nurses. Formulate and implement emergency drill plans, organize and carry out practical emergency drills, encourage diversified, efficient and regular emergency drills, focus on strengthening emergency drills for major disasters and accidents, and revise and improve emergency plans in a timely manner according to the drill.

### Electronic supplementary material

Below is the link to the electronic supplementary material.

Supplementary Material 1

## Data Availability

Data is provided within the manuscript or supplementary information files.

## References

[1] Dai Yaping. The Public Health emergencies regulations urgently need to be amended [J]. China Surv Des, 2020 (06): 23–5.

[2] Bansal VK, Dobie KH, Brock EJ. Emergency response in the ambulatory surgery Center. Anesthesiol Clin. 2019;37(2):239–50. 10.1016/j.anclin.2019.01.012. Epub 2019 Mar 16. PMID: 31047127. 31047127 10.1016/j.anclin.2019.01.012

[3] Ministry of Emergency Management --Institute for Disaster Reduction and Emergency Management, Ministry of Education. Global Disaster Data Platform. https://www.gddatcn/newGlobalWeb/#/DisasBrowse. [Last accessed on 2023.08.31].

[4] Department of International Cooperation and Relief. Circular of the state council on the planning of the national emergency response system for the fourteenth five-year plan. Natl Dev [2021] 36,https://www.mem.gov.cn/gk/zfxxgkpt/fdzdgknr/202208/t20220818_420530.shtml

[5] Yeager VA, Menachemi N, McCormick LC, Ginter PM. The nature of the public health emergency preparedness literature 2000–2008: a quantitative analysis. J Public Health Manage Pract. 2010;16(5):441–9. 10.1097/PHH.0b013e3181c33de4 20689394

[6] Kang Jie Y. Investigation on management status of disinfection supply center in 2271 hospitals in China [J]. Chin J Hosp Infectiology. 2019;34(06):950–3.

[7] Huang Qinghua F, Ximei LF, Wei, et al. Investigation of sterilizing equipment in the disinfection supply center of Chengdu Regional Hospital [J]. Chin J Antiseptics. 2020;37(7):510–2. 10.11726/j.issn.1001-7658.2020.07.010 10.11726/j.issn.1001-7658.2020.07.010

[8] Kunii Y, Usukura H, Otsuka K, Maeda M, Yabe H, Takahashi S, Tachikawa H, Tomita H. Lessons learned from psychosocial support and mental health surveys during the 10 years since the Great East Japan Earthquake: establishing evidence-based disaster psychiatry. Psychiatry Clin Neurosci. 2022;76(6):212–21. Epub 2022 Mar 1. PMID: 35137504; PMCID: PMC9314661. 35137504 10.1111/pcn.13339 PMC9314661

[9] Liu W, Xu H, Ma Y, Xuan X. Effect of scenario simulation emergency drills training in outpatient risk management [J]. Corps Med. 2022;20(04):70–2.

[10] Labrague L, Hammad K, Gloe D, McEnroe-Petitte DM, Fronda D, Obeidat A, et al. Disaster preparedness among nurses: a systematic review of literature. Int Nurs Rev. 2018;65(1):41–53. doi: 10.1111/inr.12369. [Medline: 28295314]. 28295314 10.1111/inr.12369

[11] Huang, Huan. Zhang Yibo. Advances in research on factors influencing nurse disaster preparedness and response strategies [J]. Occup Health. 2021;37(14):2004–7.

[12] Li Wentao A, Libin Y, Huiru, et al. Study on Disaster Medical Rescue Preparedness of Urban Community Health Service Center in Jilin Province [J]. Chin Gen Med. 2013;16(17):1474–6.

[13] Chen ZP. Research on status quo and improvement of public health emergency response capacity in Xuancheng City [D]. Anhui medical university, 2023. 10.26921 /.

[14] Office of emergency management, general office of the state council. Emergency management guidelines. https://www.mem.gov.cn/. [Last accessed on2023.08.31].

[15] Yumei Liu L, Wu K, Wang, et al. Medical Students Respond to Public Health Emergencies with confidence investigation [J]. Adv Mod Biomed. 2013;13(2):350–2.

[16] Wang Dongye Z, Wanli X, Shaomei, et al. Investigation and Analysis of Emergency Capacity of Community nurses in Wenzhou City [J]. J Nurs. 2016;31(4):82–4.

[17] Zhang S-L, Wang R, Li Z. Investigation on the status of emergency preparedness plan management in Zhejiang Province [J]. Industrial Safety and Environmental Protection, 2013, 39 (12): 76–80. 10.3969/j.issn.1001-425X. 2013.12.025.

[18] Li M, Wei H, Bo S, et al. Development of grass-roots modular combat trauma emergency kit [J]. J Practical Med. 2019;36(11):1055–6. 10.14172/j.ssn1671-4008.2019.11.030 10.14172/j.ssn1671-4008.2019.11.030

[19] Asadi-Pooya AA, Hosseini SA. Seizure rescue medications are missing from in-flight medical emergency kits. Epilepsy Behav. 2022;137(Pt A):108976. doi: 10.1016/j.yebeh.2022.108976. Epub 2022 Nov 10. PMID: 36370544. 10.1016/j.yebeh.2022.108976 36370544

[20] Ding SS. Design and application of thermal emergency kit for neonatal transport [J]. Chin J Rural Med 2023. 2023;30(1):6177. 10.3969/j.issn.1006-5180 10.3969/j.issn.1006-5180

[21] Chen, Chunyu. Chen Pei Juan. Chemotherapeutic drug spill response kit [J]. Global Chin Med. 2015;8(S1):138.

[22] Chen B, Hao H, Min G et al. Investigation of emergency medical material management in disinfection supply centers [J]. Journal of Nurse Development, 2021, 36 (24): 2275–2279. 10.16821/j.cnki.hsjx. 2021.24.016.

[23] Liu Y, Ying L, Lijun Z, Hanjie L. Investigation into the emergency response capacity of medical personnel of tertiary medical institutions in Wuhan [J]. South China Prev Med. 2023;49(06):724–8.

[24] Xingqiong G. Correlation between emergency-response ability and attitude of clinical nurses in Yunnan province [J]. Clin Nurs China. 2022;14(09):537–41.

[25] Jiang NN, Xuan Y. Kessel Abduk Jemmu. Investigation and analysis of emergency response capacity of community health workers in Urumqi city. J Xinjiang Med Univ. 2023;46(02):264–8.

[26] Smithers B, Tenhunen ML. Planning and Implementing Disaster drills for undergraduate nursing students. Nurs Educ Perspect. 2020 Mar/Apr;41(2):130–1. 10.1097/01.NEP.0000000000000430 30407991

[27] Brinjee D, Al Thobaity A, Almalki M, Alahmari W. Identify the Disaster Nursing Training and Education Needs for Nurses in Taif City, Saudi Arabia. Risk Manag Healthc Policy. 2021;14:2301–10. 34104020 10.2147/RMHP.S312940 PMC8180276

