# Supplementary material for: Emergency preparedness in the central sterile supply department: a multicenter cross-sectional survey
Source: BMC Emerg Med. 2024 Jul 29;24:133. doi: 10.1186/s12873-024-01053-3 (PMC11287826; doi:10.1186/s12873-024-01053-3)
Supplement: Supplementary file 1 — Supplementary Material 1 [file 12873_2024_1053_MOESM1_ESM.docx]

**Investigation on emergency drill status and emergency ability of nurses in China Disinfection Supply Center**

Dear Supply expert: Hello!

The disinfection supply center is the key department of hospital infection management, which is not only responsible for the cleaning, disinfection, packaging, sterilization and distribution of reusable medical devices in the hospital, but also the key department of hospital infection control. CSSD emergencies mainly include emergencies in the internal and external environment of the department, including positive biological indicator monitoring, toxic gas leakage, chemical agent leakage, sudden infectious diseases of unknown cause, occupational exposure, fire, earthquake, etc., which are characterized by suddenness, urgency, harm and uncertainty. The rapid response and efficient handling of emergencies in CSSD can ensure the life safety of patients and employees, reduce the loss of property, and maximize the timeliness and effectiveness of sterile supplies. Emergency drill is a very complex process, its purpose is to achieve through the drill in case of emergency or emergencies staff have the real response ability and emergency operation level, as a guarantee means to improve the quality of the whole personnel team, its importance is self-evident.

At present, there are few reports on CSSD emergency drills in hospitals at all levels in provinces. This research team conducted a survey on CSSD emergency drills and the emergency ability of CSSD nurses, with a view to improving the CSSD emergency management system and improving the emergency handling ability of CSSD emergencies in combination with the current practical work needs.

All information relating to your identity in this online survey will not be asked or recorded. Your participation in this survey will not be at any risk. If you agree to complete this survey, please read on and fill it out. However, if you feel uncomfortable at any time, you can withdraw from this survey at any time.

【 Form filling instructions 】

The questionnaire consists of five parts: the basic information of you and your organization, the emergency preparedness questionnaire, the emergency drill questionnaire, the psychological emergency attitude scale for public health emergencies and the emergency response capacity scale for public health emergencies. Please fill in the first, second and third parts according to the actual situation of your institution. If you choose other parts, please fill in the specific content. There is no right or wrong answer in Parts 4 and 5. Please choose the one you think is most appropriate. The questionnaire is expected to take 10-15 minutes of your time. We will keep the information you fill in strictly confidential, and ensure that the information obtained is only used for scientific research, please send back within 2 weeks after receiving the email. I would like to express my sincere thanks to you and sincerely look forward to your valuable comments and suggestions!

Investigation unit: Pulp washing and Disinfection Supply Center of West China Hospital, Sichuan University

Research group contact: Chen Hui

2023.03.03

**I. Basic information of participants and medical institutions**

1. Your gender

male

female

2. Your age

3. Your highest level of education

Technical secondary school (high school) and below

Junior college

Undergraduate course

Master's degree or above

4. Your title

Nurse

Nurse practitioner

Supervisor nurse

Deputy chief nurse

Chief nurse

5. Your position

Head nurse

Area leader

other

6. The address of your hospital

Detailed address of province, city, district/county

7. Have you had any emergency rescue experience in your career

is

no

8. Whether to regularly participate in in-hospital or in-department emergency training

is

no

9. Whether to participate in in-hospital or in-department emergency drills

is

no

10. Main ways to acquire emergency knowledge ()

The press

Radio and television

Weibo, wechat and other media means

Institutional training

Courses during school

Self-reading books

other

11. Number of staff at your disinfection supply center ()

>150 people

100-150 people

80-100 people

50-80 people

30-50 people

<30 people

12. The level of your hospital

Secondary hospital

Tertiary hospital

other

13. The type of hospital you are in

General hospital

Stomatological hospital

Cardiovascular hospital

Ent Hospital

Cancer hospital

Mental health center

Maternal and child health care hospital

other

14. The number of beds in your hospital

<100 sheets

100-200

200-300

300-500

500-800 copies

800-1200 copies

>1200 photos

15. The daily number of operations performed at your hospital

<50 sets/day

50-100 units/day

100-300 units/day

>300 units/day

**Ⅱ. CSSD Emergency Preparedness Questionnaire**

1. Does your department have an emergency management leadership team?

is

no

2. Does your department have a relevant emergency management system?

is

no

3. If yes, what are the emergency management systems formulated by your department? (multiple choice)

Occupational protection management system

Monitoring system

Equipment management and maintenance system

Tobacco control management system

Ethylene oxide sterilizer safety management system

Highly hazardous chemicals management system

other

4. Does your department have an emergency plan?

is

no

5. If yes, what emergency plans have been developed by your department (multiple choice)?

Fire emergency plan

Ethylene oxide leak emergency plan

Emergency plan for positive biological monitoring

Emergency plan for sudden infectious disease of unknown cause

Emergency plan for occupational exposure

Earthquake emergency plan

Flood emergency plan

Emergency plan for information system failure

Emergency plan for large medical equipment failure

Chemical spill emergency plan

Emergency plan for sudden power failure

Emergency plan for sudden water outage

other

6. Does your department regularly update or revise the emergency plan?

is

no

7. Do you have emergency supplies in your department?

is

no

8. What kind of emergency supplies?

Personal protective materials

Disinfection and sterilization materials

Medical supplies

other

9. Does your department have job responsibilities for emergencies?

is

no

10. Is there a special person in your department responsible for emergency drills?

is

no

11. Is an emergency drill plan prepared before the drill starts?

is

no

12. Does your department conduct a final evaluation and summary of the results of the emergency drill?

is

no

14. Does the hospital where you work offer training on emergency response?

is

no

15. Frequency of training?

frequently

Now and then

seldom

scarcely

never

16. Will the effectiveness of the training be evaluated?

is

no

17. As a paramedic, what emergency related training do you want? (multiple choice)

Disaster management

Information transfer in disasters and crises

Psychological care after trauma

Basic life support

Advanced life support

Pre-test triage

other

**Ⅲ. CSSD emergency exercise questionnaire**

1. Contents of emergency drill

Single emergency drill - A drill for one of the emergency response functions in the emergency plan

Integrated emergency drills - Drills for many or all of the emergency response functions in the emergency plan

2. Items of emergency drill

Fire emergency drill

Ethylene oxide leak emergency drill

Emergency drill for positive biological monitoring

Emergency drill for infectious disease of unknown cause

Emergency training for occupational exposure

Earthquake emergency drill

Flood emergency drill

Information system fault emergency drill

Large medical equipment failure emergency drill

Chemical spill emergency drill

Steam leak emergency plan

Sudden power failure emergency drill

Emergency drill for sudden water outage

other

3. Forms of emergency drills

Desktop drill - Emergency drill activities for emergency scenarios, using drawings, sand tables, flow charts, computer simulation, video conferencing and other auxiliary means to conduct interactive discussion and deduction

Actual combat exercise: According to the accident scenario, select equipment, facilities, devices or places in production and business activities, use all kinds of emergency equipment, equipment and materials, and complete the process of real emergency response through decision-making actions and practical operations

4. Frequency of emergency drills

Once/month

Once/every quarter

Once/every six months

Once/every year

5. Organization form of emergency drill

sponsor

Do sth. jointly

attend

6. Duration of emergency drill

<30min

30-60min

60-90min

90-120min

>120min

7. Personnel involved in emergency drills

All department members are involved

70%-90% of the department staff are involved

50% to 70% of the department staff participated

30%-50% of department staff participate

Department participants <30%

8. Is relevant technology (3D visual simulation system, accident consequence model technology, etc.) used to support the emergency drill?

is

no

9. Obstacles to the implementation of emergency drills

Financial difficulty

Supplies and equipment are lacking

Lack of guidelines for emergency drills

Not enough attention from superiors

Coordination between regions is difficult

Lack of institutional guarantee

Lack of site security

other

**Ⅳ. Psychological emergency attitude Scale for public health emergencies**

1. I am terrified of public health emergencies

Totally agree 1 point

Agree 2 points

Not sure 3 points

4 points for disagreement

Totally disagree 5 points

2. I am familiar with public health emergencies

Completely disagree 1 point

Disagree 2 points

Not sure 3 points

Agree 4 points

Totally agree with 5 points

3. I am willing to learn about public health emergencies

Completely disagree 1 point

Disagree 2 points

Not sure 3 points

Agree 4 points

Totally agree with 5 points

4. It is necessary to carry out courses and training related to public health emergencies

Completely disagree 1 point

Disagree 2 points

Not sure 3 points

Agree 4 points

Totally agree with 5 points

5. It is necessary to carry out emergency drills for public health emergencies

Completely disagree 1 point

Disagree 2 points

Not sure 3 points

Agree 4 points

Totally agree with 5 points

6. I will keep an eye on the prevalence of local infectious diseases

Completely disagree 1 point

Disagree 2 points

Not sure 3 points

Agree 4 points

Totally agree with 5 points

7. I will follow the development of public health emergencies

Completely disagree 1 point

Disagree 2 points

Not sure 3 points

Agree 4 points

Totally agree with 5 points

8. It is necessary to have a fire extinguisher at home

Completely disagree 1 point

Disagree 2 points

Not sure 3 points

Agree 4 points

Totally agree with 5 points

9. It is important to have knowledge of public health emergencies

Completely disagree 1 point

Disagree 2 points

Not sure 3 points

Agree 4 points

Totally agree with 5 points

10. I am willing to participate in the rescue work of public health emergencies

Completely disagree 1 point

Disagree 2 points

Not sure 3 points

Agree 4 points

Totally agree with 5 points

**Ⅴ. Public health emergency Response Capacity Scale**

1. I can identify common public health emergencies

Well done 5 points

Well done 4 points

Do average 3 points

That's two points off

Did a very poor 1

2. I am able to grasp the concept of public health emergencies and relevant laws, regulations and regulations

Well done 5 points

Well done 4 points

Do average 3 points

That's two points off

Did a very poor 1

3. I can master the reporting knowledge and registration process of public health emergencies

Well done 5 points

Well done 4 points

Do average 3 points

That's two points off

Did a very poor 1

4. I can master the on-site rescue knowledge of public health emergencies

Well done 5 points

Well done 4 points

Do average 3 points

That's two points off

Did a very poor 1

5. I can master the public health emergency response plan formulated by the unit

Well done 5 points

Well done 4 points

Do average 3 points

That's two points off

Did a very poor 1

6. I can master on-site rescue techniques (cardiopulmonary resuscitation, bandaging, etc.)

Well done 5 points

Well done 4 points

Do average 3 points

That's two points off

Did a very poor 1

7. I can master the technology of personal safety protection

Well done 5 points

Well done 4 points

Do average 3 points

That's two points off

Did a very poor 1

8. I can carry out correct triage, placement and transport according to the condition of patients

Well done 5 points

Well done 4 points

Do average 3 points

That's two points off

Did a very poor 1

9. I can use ECG monitor, negative pressure aspirator, simple breathing apparatus and other first aid equipment

Well done 5 points

Well done 4 points

Do average 3 points

That's two points off

Did a very poor 1

10, I can quickly, accurately and timely record the patient's condition changes and take relevant measures

Well done 5 points

Well done 4 points

Do average 3 points

That's two points off

Did a very poor 1

11. I can master the prevention and isolation measures of common infectious diseases

Well done 5 points

Well done 4 points

Do average 3 points

That's two points off

Did a very poor 1

12. I can master communication skills and establish good interpersonal relationships

Well done 5 points

Well done 4 points

Do average 3 points

That's two points off

Did a very poor 1

13. I can assist with vaccinations and prophylactic medication

Well done 5 points

Well done 4 points

Do average 3 points

That's two points off

Did a very poor 1

14. I can provide effective nursing care for patients with infectious diseases and suspected patients

Well done 5 points

Well done 4 points

Do average 3 points

That's two points off

Did a very poor 1

15, I can well assist in carrying out the disinfection of the epidemic area

Well done 5 points

Well done 4 points

Do average 3 points

That's two points off

Did a very poor 1

16. I can identify the psychological or emotional needs of patients and community residents during public health emergencies

Well done 5 points

Well done 4 points

Do average 3 points

That's two points off

Did a very poor 1

17. I have the ability to provide patients and community members with the psychological care and intervention they need

Well done 5 points

Well done 4 points

Do average 3 points

That's two points off

Did a very poor 1

18. I am capable of carrying out health education on public health emergencies for disaster victims

Well done 5 points

Well done 4 points

Do average 3 points

That's two points off

Did a very poor 1.
